# Transcriptome Analysis of Dendrobine Biosynthesis in *Trichoderma longibrachiatum* MD33

**DOI:** 10.3389/fmicb.2022.890733

**Published:** 2022-08-01

**Authors:** Qi Jia, Lina Wang, Xu Qian, Hui Jin, Fuxing Shu, Surendra Sarsaiya, Leilei Jin, Jishuang Chen

**Affiliations:** ^1^College of Biotechnology and Pharmaceutical Engineering, Nanjing Tech University, Nanjing, China; ^2^Bioresource Institute for Healthy Utilization, Zunyi Medical University, Zunyi, China; ^3^Key Laboratory of Basic Pharmacology of Ministry of Education and Joint International Research Laboratory of Ethnomedicine of Ministry of Education, Zunyi Medical University, Zunyi, China

**Keywords:** dendrobine, *Trichoderma longibrachiatum* MD33, differentially expressed genes, MVA pathways, P450 family

## Abstract

Dendrobine is a representative component of *Dendrobium nobile*, and its pharmacological effects have been extensively studied. *Trichoderma longibrachiatum* MD33 was isolated from the stem of *Dendrobium nobile* which can produce dendrobine. In order to understand the effect of Methyl Jasmonate (MeJA) on the production of dendrobine, transcriptome analysis was performed after MeJA treatment in the MD33 and control groups. The dendrobine production of MeJA (20 μmol/L) treatment group was 44.6% higher than that of control. In this study, the RNA sequencing technology was applied, a total of 444 differentially expressed genes (DEGs) in the control and MeJA treatment groups, including 226 up-regulated genes and 218 down-regulated genes. The Kyoto Encyclopedia of Genes and Genomes annotation showed that numbers of DEGs were associated with the putative alkaloid biosynthetic pathway in *T Trichoderma longibrachiatum* MD33. Several MVA pathway enzyme-coding genes (isopentenyl-diphosphate Delta-isomerase, iphosphomevalonate decarboxylase and farnesyl diphosphate synthase) were found to be differentially expressed, suggesting an active precursor supply for alkaloid biosynthesis after MeJA treatment, in other wise, dendrobine may synthesis through the MVA pathway in MD33. Numerous MeJA-induced P450 family genes, aminotransferase genes and methyltransferase genes were identified, providing several important candidates to further elucidate the dendrobine biosynthetic pathway of *T. longibrachiatum* MD33. Furthermore, several MeJA-induced transcription factors (TFs) encoding genes were identified, suggesting a complex genetic network affecting the dendrobine in *T. longibrachiatum* MD33. These findings reveal the regulation mechanism underlying the MeJA-induced accumulation of dendrobine in *T. longibrachiatum* MD33.

## Introduction

Dendrobine is a sesquiterpene alkaloid mainly found in the medicinal orchid *Dendrobium nobile*, which is considered to possess therapeutic properties. It is a representative component of the *D. nobile* species (Zhang et al., 2021; Li et al., 2022). Dendrobine possesses numerous of pharmacological properties, including analgesic and antipyretic properties, the ability to regulate blood glucose and blood pressure (Bai et al., 2020), antitumor activity (Song et al., 2018), and inhibition of cardiovascular system and gastrointestinal tract (Mou et al., 2021). It has also demonstrated promising therapeutic effects in Alzheimer’s disease (Nie et al., 2018; Xiong et al., 2021). Dendrobine significantly reduces the ischemic damage caused by oxygen-glucose deprivation/reperfusion (OGD/RP) to brain slices and has a major protective effect on primary neurons of OGD/RP (Liu et al., 2021). However, given the slow growth rate of the *D. nobile* species, dendrobine is seldom produced in large amounts. The quantity of *D. nobile* dendrobine available is insufficient to meet the present industrial and research needs (Jiang et al., 2018; Zhang Y. et al., 2018).

In 1980s, plant endophytic fungi were found and recognized. These fungi live in the tissues and organs of plants without causing evident illness. Paclitaxel producing short leaf Taxus was the first endophytic fungi found in medical plants, after which different kinds of endophytic fungi were found (Stierle et al., 1993). Among these, the taxol content of endophytic fungus ne-32 (*Pestalotipsis microspora*) obtained from Taxus is more than 1,000 times higher than that of the host (Strobel et al., 1996), Thereafter, endophytic fungi producing camptothecin, podophyllotoxin, vinblastine, huperzine A, ginsenoside, and Dendrobium polysaccharide were consecutively isolated. There are at least one million species of plant endophytic fungi (Fagan, 2002) and some medicinal plant endophytic fungi produce a certain amount of “plant-derived” bioactive substances. Hence, we researched and isolated several dendrobine-producing endophytic fungi from *D. nobile*, of which T. *longibrachiatum* MD33 produced the highest amount of dendrobine (Sarsaiya et al., 2019b).

A dendrobine metabolic pathway in the Dendrobium plant has been defined based on transcriptome sequencing and data analysis. The mechanism by which the mycorrhizal fungus MF23 increased dendrobine content in *D. nobile* seedlings was analyzed using the transcriptome, the dendrobine synthetic pathway was proposed for the first time, and key modifying enzymes such as cytochrome P450, aminotransferase, and methyltransferase were identified (Li et al., 2017). Chen et al. (2019) determined the expression levels of P450 family, aminotransferase, and methyltransferase genes activated by MeJA. MeJA induces a significant number of transcription factor-coding genes. The effector stress culture is a very useful research model for alkaloid active compounds. Examining the link between products and gene expression under regulated settings enables direct identification of functional genes or gene clusters involved in alkaloid production pathway.

The phytohormone (MeJA) has been extensively used to stimulate secondary metabolite production in medicinal plants (Xiaori et al., 2018). MeJA acts as a signaling molecule various secondary metabolites, notably alkaloids (Zhang X. N. et al., 2018). However, the mechanism by which MeJA induces accumulation of sesquiterpene alkaloids in *T. longibrachiatum* MD33 remains unclear. Numerous differentially expressed genes (DEGs) were identified in this study to clarify the regulatory mechanism driving the accumulation of sesquiterpene alkaloids in *T. longibrachiatum* MD33 triggered by MeJA, which may provide a source of dendrobine.

## Materials and Methods

### Fungal Strain, Culture Conditions, and Methyl Jasmonate Treatment

**FIGURE 1:**
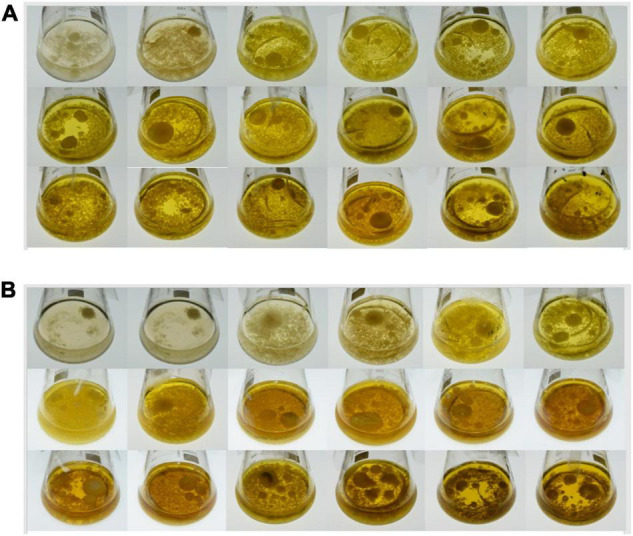
The growth of *Trichoderma longibrachiatum* MD33 MeJA treatment group and control group (18 days). **(A)** MD33 growth in MeJA treatment group. From the third day, the color of liquid gradually deepened, from light yellow to brown, the biomass of MD33 increased significantly from the second day, in the seventh day, the biomass reached the maximum and remained basically unchanged. **(B)** MD33 growth in Control group. In the first 4 days, the biomass remained at a low level and the color of liquid was limpid, from the fifth day to the eighteenth day, the color gradually changed from light yellow to brown. From the eighth day, the biomass reached the maximum.

The *Trichoderma longibrachiatum* MD33 was isolated from stem segments of a single wild *D. nobile* (Sarsaiya et al., 2019a,2020b) and stored at the laboratory of Nanjing Tech University’s Institute of Bioresources Engineering. After activating the stored fungus for 48 h, it was transferred to PD medium and cultivated at 28°C for 36 h. Thereafter 0.1180 g MeJA (95%) was completely dissolved in pure ethanol, transferred to a 10 ml volumetric flask and dilute with deionized water to 50 mmol/L MeJA. Methyl Jasmonate (MeJA) was added to PD medium at 0, 5, 20, and 35 μmol/L (250 ml Erlenmeyer flask, 100 ml PD medium), eight bottles per group. The pH value at the beginning was neutral. The block at the colony’s edge where the new hypha forms was removed and put into each flask using a 6 mm punch. The culture conditions used with the strain were neutral pH, 28°C, 120 rpm rotation speed, and 15 days (Sarsaiya et al., 2020a). The growth of MD33 were shown in Figure 1. There were three biological replicates for each group.

### Determination of Dendrobine in MD33

Five bottles of *Trichoderma longibrachiatum* MD33 were used for dendrobine extraction and detection. The following steps were used: The fungi cultured in shake flasks were filtered, dried to constant weight at 45°C, and ground to a powder. Thereafter 50 ml chloroform was added to the powder, transferred to a separation funnel for static stratification after 30 min of ultrasonic extraction, distilled and the lower solvent layer was dried under reduced pressure (rotary evaporator SHZ-III model). The dried powder was dissolved in 5 ml methanol centrifuged at 1,000 rpm for 15 min. Thereafter 1 ml solution was filtered via a 0.22 μm filter membrane, and a sample was injected for detection.

### Analysis of Dendrobine in MD33 by LC/MS

The LC-MS technique was used to characterize non-volatile and thermally fragile compounds. The detection of dendrobine was performed using a UHPLC system (Thermo Fisher Scientific Dionex Ultimate 3000, Golden Valley, Minnesota, United States) with a column (150 × 2.1 mm,1.9 μm) and a mobile phase consisting of 0.1% formic acid: acetonitrile at 95:5 (v/v) with a flow rate of 0.3 ml/min, column temperature of 40°C, feed volume 2 μl, a sheath gas flow rate of 35 arbitrary units, an auxiliary gas flow rate of 15 arbitrary units, a spray voltage of 3.5 kV, a capillary temperature of 350°C, an aux gas heater temperature of 300°C, the detection range was 100–1,500 m/Z, the scanning mode was full scan/positive ion scan, and the resolution was MS full scan 70,000 full width at half maxima (FWHM).

**FIGURE 2:**
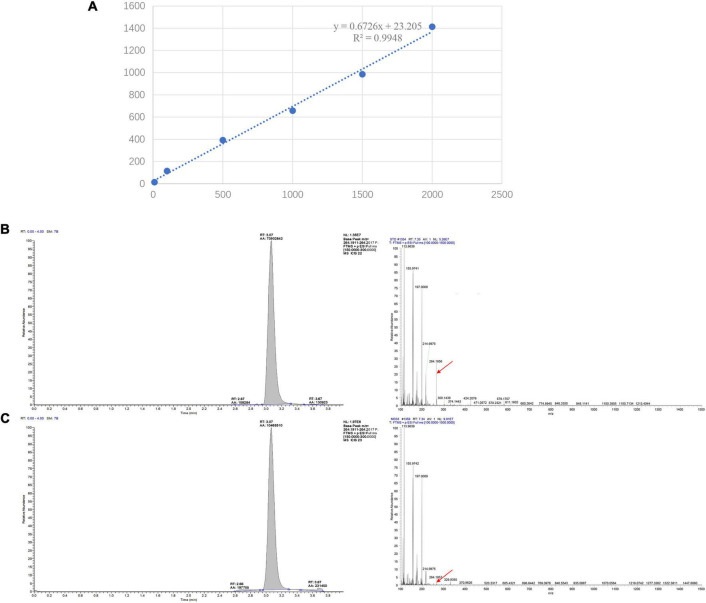
LC-MS/MS chromatographs for the detection of dendrobine. **(A)** LC-MS detection standard curve of Dendrobine. The regression curve equation of dendrobine is y = 0.6726x + 23.205, *R*^2^ = 0.9948. **(B)** *T. longibrachiatum* MD33 intracellular dendrobine. **(C)** *Dendrobium nobile* stem dendrobine (molecular weight: 264.195).

To provide a scientific rational for MeJA-induced dendrobine accumulation in MD33, a standard curve was established and the contents of dendrobine were evaluated under control and MeJA conditions. For the standard curve, 1.00 mg of dendrobine standard was accurately weighed, dissolved in methanol to prepare 1 g/L mother liquor, and then diluted into standard solutions at concentrations of 1, 10, 100, 500, 1,000, and 1,500 ng/L. The linear regression equation and standard curve were constructed using the LC-MS detection results. The regression curve equation of dendrobine is y = 0.6726x + 23.205, *R*^2^ = 0.9948 (Figure 2A). Each sample detected for three times.

### RNA Isolation and Construction of cDNA Libraries

The cultivated *Trichoderma longibrachiatum* MD33 was treated according to the LC/MS findings (Step 2.1 and Step 2.2). Three flasks from each group were randomly selected and filtered. Prior to RNA extraction, the hypha samples were snap-frozen in liquid nitrogen and stored at –80°C.

Total RNA was extracted from hypha using the RNeasy mini kit (QIAGEN, Cat. No. 74104, Germany) and genomic DNA was removed using DNase. Then RNA quality was determined by Bioanalyzer 2100 and the RNA 6000 Nano LabChip kit (Agilent, CA, United States). Only high-quality RNA sample (OD260/280 = 1.8–2.2, OD260/230 ≥ 2.0, RIN ≥ 6.5) was used A SureSelect strand-specific RNA library preparation kit from Agilent Technologies was used to generate an RNA-seq library according to the manufacturer’s procedure using 2 μg of total RNA. The Agilent 2100 Bioanalyzer and the ABI Step One Plus Real-Time PCR system was used to assess the quality of the RNA-seq library. Thereafter, sequencing was performed on the Illumina HiSeq 4000 platform. The transcriptome library was prepared by KEGENE Company (Shandong, China). There were three biological replicates for each group.

### *De novo* Assembly and Annotation

The raw FASTQ data was analyzed for quality using FastQC and filtered to remove reads with sequencing adaptors, unknown nucleotides (Nradio > 5%), and poor quality (quality scores 30). Due to the unavailability of a reference genomic sequence, *de novo* assembly was used to create the transcripts. The redundant sequences were eliminated using the CORSET software suite, and the longest unigenes were further spliced. All assembled unigenes were aligned against the non-redundant (Nr), nucleotide (Nt), protein family (Pfam), Gene Ontol-ogy (GO), SwissProt, and KOG/COG, Kyoto Encyclopedia of Genes and Genomes (KEGG) databases with a threshold of *E*-value < 10^–10^.

### Differentially Expressed Genes Analysis

In this study, the expression level of all transcripts was standardized using RSEM and Bowtie2 with default parameters. Ballgown was used for Differentially Expressed Gene (DEG) investigation. An FDR value of (0.05) and a log2FC value of 1 were used as the criteria for DEG screening. The DEGs were then analyzed for GO and KEGG enrichment.

### RNA-Seq Validation by q-RT PCR

qRT-PCR was performed to validate the accuracy of RNA-seq data. GAPDH gene was used as an internal reference. The foldchange in gene expression was calculated using the comparative Ct method (1 + E^–△△Ct^) (Pfaffl, 2001). Total RNA samples were extracted from the hyphae using RNeasy mini kit (QIAGEN, Cat: 74104, Germany). The cDNA synthesis kit (TSINGKE, TSK302M) was used to synthesize cDNA from 1 μg of RNA. For gene expression analysis, the Applied Biosystems StepOnePlus™ Real-Time PCR system was used. qRT-PCR amplification was performed in 20 μl reactions containing 10 μl 2 × TSINKE^®^ Master qPCR Mix (SYBR GREEN), 20 ng cDNA, and 0.4 μM of each primer (Supplementary Table 5). The PCR reaction conditions were: 95°C for 15 min, followed by 40 cycles of 95°C for 15 s, 60°C for 30 s. After final annealing (72°C, 5 min) and redenaturation (95°C, 30 s), a melt curve analysis was done by increasing from 60 to 95°C at 0.5°C intervals.

## Results

### Determination of Dendrobine Contents

**FIGURE 3:**
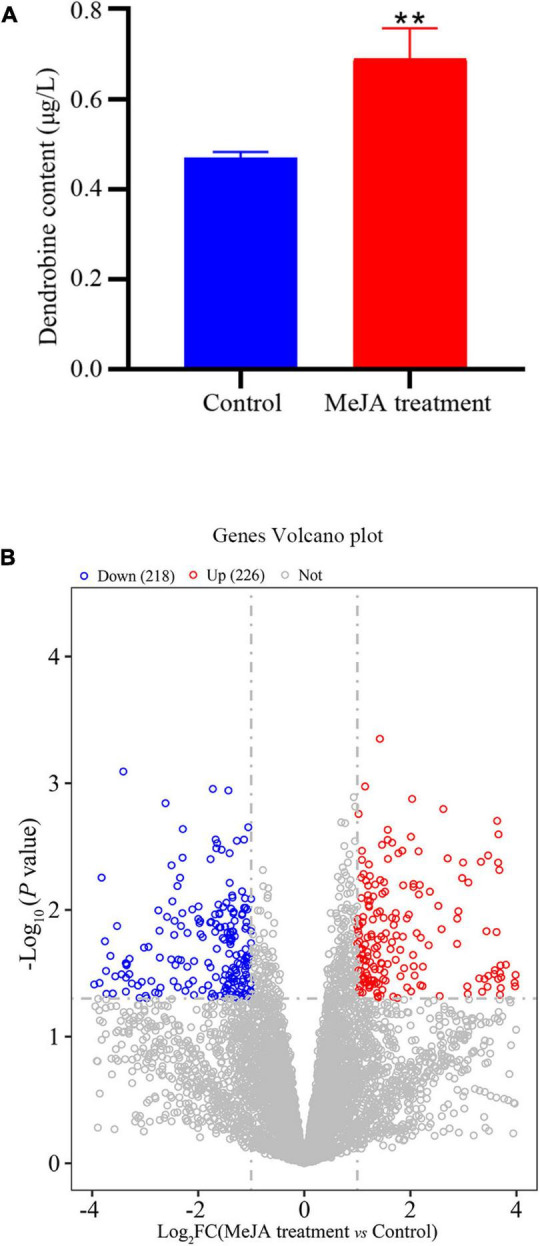
Transcriptional variation between Control and MeJA treatment groups. **(A)** In the MeJA treatment group dendrobine content increased 44.6%, **(B)** significance analysis of all DEGs between the strain MeJA treatment MD33 and Control by a volcano plot. **p < 0.01.

The LC-MS results revealed that standard chemical reference dendrobine was found at a retention time of 3.05 and a molecular weight of 264.195. From the MD33 dendrobine the dendrobine peak was recorded at 3.07 with a molecular weight of 264.195 (Figures 2B,C). LC-MS detection results showed that, the content of dendrobine in MeJA treatment groups were higher than control group. When the concentration of MeJA was 20 μmol/L, the content of dendrobine was the highest (0.68 μg/L), which increased 44.7% compared with the control group (0.47 μg/L) (Figure 3A). indicating that MeJA plays an important role in dendrobine accumulation in MD33.

### *De novo* Assembly and Unigene Annotation

Three distinct RNA-seq libraries were generated utilizing RNA from the control and MeJA treated groups. A total of 274,332,098 pure reads were isolated from 288,958,158 raw reads in this study, equal to 41.16 Gb of clean bases (Supplementary Table 1). The Q20/Q30 fraction, the N proportion, and the GC percentages were 98.73/95.64, 0.00, and 58.05%, respectively. The clean sequences were then *de novo* assembled and clustered into 21,331 (median length = 3,850 bp) and 4,477 (median length = 3,326 bp) genes, respectively, using Trinity (Supplementary Tables 2, 3). We analyzed the size distributions of transcripts and genes, respectively. For transcripts, the majority (76.22%) were > 2,000 bp in length, 23.00% were 500–2,000 bp in length, and 0.77% were 500 bp in length. For genes, 42.93% were > 2,000 bp in length, 54.75% were 500–2,000 bp on length, and 2.32% < 500 bp in length (Supplementary Table 2). All identified genes were annotated using several databases. The NR database contained 6,037 genes, the KO database contained 1,765 genes, the KEGG database contained 1,761 genes, the KO database contained 2,920 genes, the SwissProt database contained 4,885 genes, the Pfam database contained 5,666 genes, the GO database contained 4,677 genes, and the KOG database contained 5,777 genes (Supplementary Table 4).

### Gene Ontology and Kyoto Encyclopedia of Genes and Genomes Classification of Differentially Expressed Genes

**FIGURE 4:**
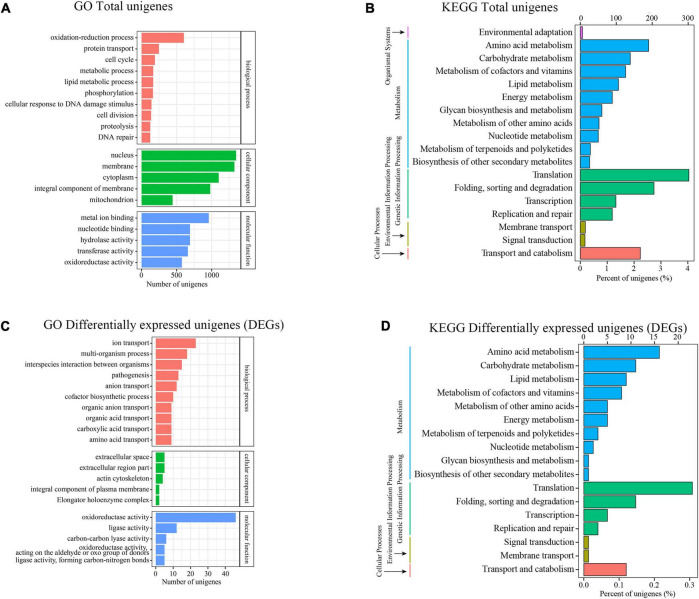
Transcriptional analysis and DEGs differential expressions between Control and MeJA treatment groups. **(A)** GO enrichment analysis (Total unigenes). **(B)** KEGG enrichment analysis (Total unigenes). **(C)** GO enrichment analysis (Differentially expressesunigenes). **(D)** KEGG enrichment analysis (Differentially expressesunigenes).

To ascertain the likely function of these DEGs in *Trichoderma longibrachiatum* MD33, we performed a Gene Ontol-ogy (GO) enrichment analysis, which revealed that these DEGs were classified into three primary categories: biological process (BP), cellular component (CC), and molecular function (MF). The terms “oxidation-reduction process,” “protein transport,” and “cell cycle” were the most frequently used in the biological process category; “nucleus,” “membrane,” and “cytoplasm” were frequently used in the cellular component category; and a large percentage of genes were associated with “metal ion binding,” “nucleotide binding,” and “hydrolase activity” in the molecular function category (Figure 4A). Moreover 1,761 genes were classified into 18 Kyoto Encyclopedia of Genes and Genome (KEGG) secondary pathways. The majority of pathways were classified as “Metabolism” (866 genes) or “Genetic Information Processing” (695 genes). Moreover, 190 genes belonged to the “amino acid metabolism” pathway, 139 genes belonged to the “carbohydrate metabolism” pathway, 126 genes belonged to the “metabolism of cofactors and vitamins” pathway, 106 genes belonged to the “lipid metabolism” pathway, and 28 genes belonged to the “metabolism of terpenoids and polyketides” pathway in metabolism, 302 genes belonged to the “translation” route in “Genetic Information Processing,” whereas 205 genes belonged to the “folding, sorting, and degradation” pathway (Figure 4B).

### Differentially Expressed Genes After Methyl Jasmonate Treatment

The DEGs that increased considerably after MeJA treatment were regarded as MeJA-regulated genes (Supplementary Table 6). We performed a significance analysis on the DEGs and displayed the results using a volcano diagram. Notably, 444 DEGs were identified between the control and MeJA treatment groups. Among these significant DEGs, 226 (50.9%) were up-regulated, while the remaining 218 (49.1%) were down-regulated (Figure 3B). The examination of GO enrichment revealed that the most enriched GO keywords were “oxidoreductase activity,” “ion transport,” “multi-organism,” “interspecies interaction between organisms,” “pathogenesis,” “anion transport,” and “ligase activity” (Figure 4C). Additionally, genes were allocated to several KEGG metabolic pathways (Supplementary Table 4). Enrichment analysis showed that the significantly enriched KEGG pathways were “Translation,” “Amino acid metabolism,” “Folding,” “sorting and degradation,” “Carbohydrate metabolism,” “Transport and catabolism,” “Lipid metabolism,” “Metabolism of cofactors and vitamins” (Figure 4D).

### Differentially Expressed Genes Involved in Secondary Metabolite Biosynthesis

The DEGs analysis of secondary metabolites revealed that, 10 clusters were up-regulated compares to the control group. These clusters are involved in secondary metabolite production and degradation, caffeine metabolism, glucosinolate biosynthesis, and novobiocin biosynthesis. There was a significant difference in Phenylpropanoid production (*p* < 0.05), and the function of the divergent gene may be beta-glucosidase. The unchanged clusters were involved in the synthesis of tropane, piperidine, and pyridine alkaloid (Supplementary Table 5).

### Expression Changes of Genes in MVA Pathway

**FIGURE 5:**
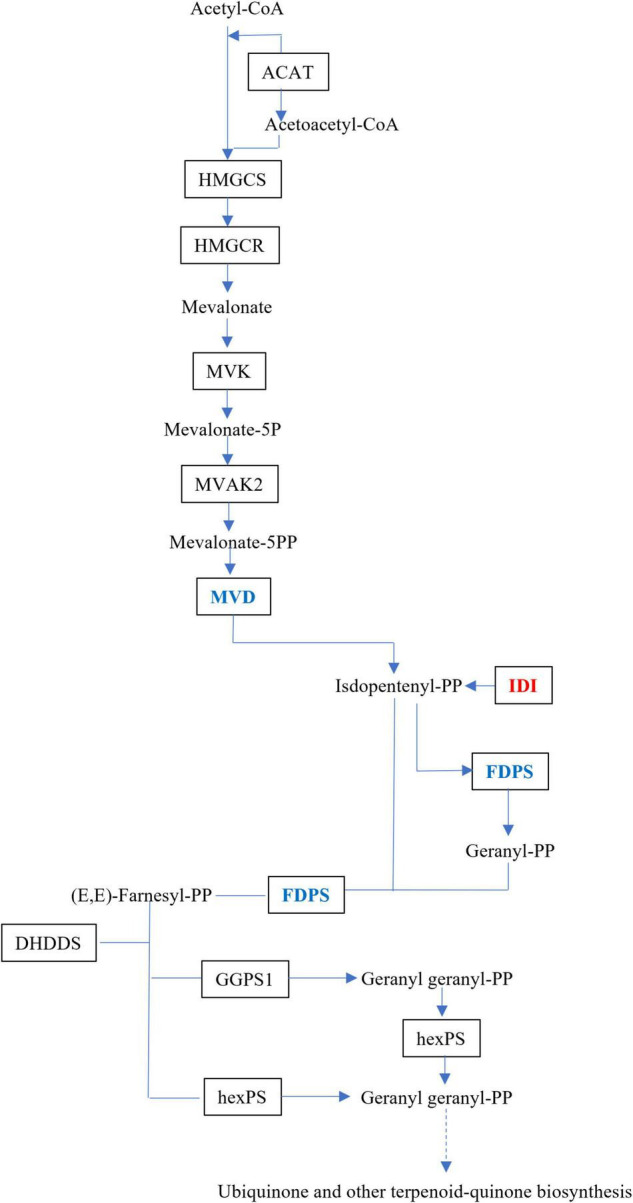
Schematic illustration of MVA biosynthetic pathways in MD33. Abbreviations, as quoted in the figure, are as follows. MVD, diphosphomevalonate decarboxylase; IDI, isopentenyl-diphosphate Delta-isomerase. Expression changes of the genes associated with MVA pathway in the MeJA treatment group and Control. Red indicates up-regulated genes and green indicates down-regulated genes.

The upstream biosynthesis routes for sesquiterpene intermediate products have been extensively researched, and are preserved in plants (Li et al., 2017). Dendrobium alkaloids are mainly sesquiterpene alkaloids, which are derived from MVA, and the MEP route would act as a source of isoprene units in the production of dendrobine (Chen et al., 2019). Three DEGs, isopentenyl-diphosphate Delta isomerase (IDI), dishomevalonate decarboxylase (MVD), and farnesyl diphosphate synthase (FDPS), were mapped to the MVA pathway in this study (Figure 5).

### Comparison of the Expression Levels of P450 Family Methyltransferase and Aminotransferase Genes

**FIGURE 6:**
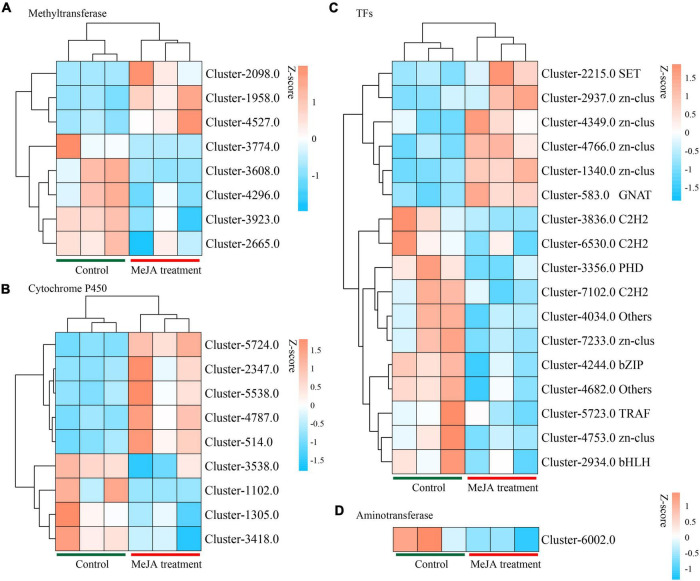
Heat map analysis of genes in backbone post-modification. **(A)** Heat map analysis of methyltransferases. **(B)** Heat map analysis of Cytochrome P450 family. **(C)** Heat map analysis of TFs. **(D)** Heat map analysis of Aminotransferase.

By examining the DEGs, we identified 51 potential P450 unigenes, nine of which were novel. MD33 with and without MeJA treatment is shown in Table 1. Cluster-4787.0, Cluster-514.0, Cluster-2347, Cluster-5538.0, and Cluster-5724.0 were found to be up-regulated, while Cluster-1305.0, Cluster-3418.0, Cluster-1102.0, and Cluster-3538.0 were found to be down-regulated (Figure 6 and Table 1).

**TABLE 1:** The comparation of P450, TFs, aminotransferases and methyltransferases between control and MeJA treatment MD33 groups.

	GeneID	Control_1	Control_2	Control_3	MeJA treatment MD33_1	MeJA treatment MD33_2	MeJA treatment MD33_3	logFC	*P*-value	Regulation	Family
P450	Cluster-4787.0	50	26	8	158	295	387	3.33	0.004175	Up	
	Cluster-514.0	172	227	143	381	499	682	1.29	0.011896	Up	
	Cluster-2347.0	163	245	177	433	732	1,187	1.69	0.01315	Up	
	Cluster-1305.0	552	511	914	487	257	389	–1.01	0.014699	Down	
	Cluster-3418.0	213	229	324	165	54	156	–1.36	0.016981	Down	
	Cluster-5538.0	34	65	43	109	152	276	1.63	0.020394	Up	
	Cluster-5724.0	34	33	30	68	88	81	1.05	0.024677	Up	
	Cluster-1102.0	4.46	34.25	32.25	0	0	0	–5.36	0.037502	Down	
	Cluster-3538.0	77.51	74.56	87.95	41.89	71.14	32.11	–1.01	0.038602	Down	
TFs	Cluster-3356.0	79.67	42.72	43.26	0	22.22	0	–5.19	0.013616	Down	PHD
	Cluster-1340.0	46	41	57	117	142	126	1.20	0.008428	Up	zn-clus
	Cluster-4034.0	234.51	110.74	237.26	100.15	91.97	35.64	–1.60	0.013737	Down	Others
	Cluster-7102.0	83.38	35.43	78.23	6.96	20.17	37.79	–2.08	0.025371	Down	C2H2
	Cluster-2215.0	44.78	29.16	17.13	202.53	131.5	61.29	1.86	0.016285	Up	SET
	Cluster-7233.0	1896.76	924.54	2239.71	778.25	567	420.22	–1.67	0.002784	Down	zn-clus
	Cluster-4349.0	0	62.33	0	116.17	78.02	191.74	5.44	0.020901	Up	zn-clus
	Cluster-4244.0	819.06	908.17	979.47	652.44	508.58	372.06	–1.06	0.002227	Down	bZIP
	Cluster-2937.0	12.45	9.37	28.87	74.72	89.09	28.13	1.68	0.048388	Up	zn-clus
	Cluster-3836.0	248.72	473.77	93.05	0	0	46.28	–6.93	0.03241	Down	C2H2
	Cluster-4682.0	2,818	2,998	3,708	2,322	1,529	955	–1.27	0.002848	Down	Others
	Cluster-4766.0	29.49	0.1	4.61	114.06	71.25	108.8	4.18	0.009628	Up	zn-clus
	Cluster-4753.0	1967.34	1365.74	3652.47	1110.08	750.6	453.44	–1.77	0.003979	Down	zn-clus
	Cluster-2934.0	618.98	771.49	1125.37	644.83	314.54	402.6	–1.11	0.009633	Down	bHLH
	Cluster-5723.0	2481.8	2282.65	5799.43	1472.02	785.16	2639.34	–1.35	0.034829	Down	TRAF
	Cluster-6530.0	514.17	1065.67	406.1	529.74	39.92	113.44	–2.32	0.024956	Down	C2H2
	Cluster-583.0	2	10	10	39	41	57	2.59	0.014198	Up	GNAT
Aminotransferases	Cluster-6002.0	41.58	46.23	22.07	16.61	16.95	8.73	–1.53	1.989755	Down	
	Cluster-4588.0	0	26.67	126.84	345.43	36.9	142.94	3.16	0.135006	Up	
	Cluster-5638.0	0	32.68	44.05	36.81	35.98	39.55	1.85	0.221301	Up	
	Cluster-5199.0	0	0	78.73	9.46	119.24	17.83	3.03	0.272025	Up	
Methyltransferases	Cluster-3608.0	530.48	267.79	574.4	151.04	180.74	153.84	–1.64	0.002978	Down	
	Cluster-1958.0	216	149	20	793	1,105	1,554	3.47	0.003718	Up	
	Cluster-3923.0	169.87	167.04	196.96	120.98	64.18	16.91	–1.96	0.012268	Down	
	Cluster-4296.0	235.39	145.03	262.02	158.19	84.68	100.34	–1.11	0.014069	Down	
	Cluster-2098.0	0	0	0	71.8	179.82	36.7	7.08	0.017249	Up	
	Cluster-2665.0	5672.98	6007.33	7140.16	5557.26	1826.78	4206.5	–1.04	0.023372	Down	
	Cluster-3774.0	6.01	32.34	6.67	0	0	0	–4.71	0.02606	Down	
	Cluster-4527.0	114	92	74	204	177	386	1.17	0.031543	Up	

Aminotransferase and methyltransferase were investigated as two additional key enzymes involved in post-modification. Clustering analysis indicated that only one aminotransferase (Cluster-6002.0) was down-regulated (*p* < 0.05), but Cluster-4588.0 (log2 fold change = 3.16-fold), Cluster-5638.0 (log2 fold change = 1.85-fold), and Cluster-5199.0 (log2 fold change = 3.02-fold) were up-regulated after MD33 MeJA treatment (Table 1). According to the reference genome (*T. longibrachiatum* ATCC 18648) (Shi et al., 2020), Cluster-6002.0 encodes a glutamate-1-semialdehyde aminotransferase. The metabolic pathways implicated may include heme (k01845) production and porphyrin and chlorophyll metabolism (ko00860). Heme and porphyrin are pyrrole chemicals with a similar structure to dendrobine. Hence this gene may be implicated in the transaminase function during dendrobine production (Table 1).

By analyzing the DEGs, we obtained 140 putative methyltransferase unigenes, of which eight unigenes (Cluster-1958.0, Cluster-2098.0, and Cluster-4527.0 increased expression, Cluster-3608.0, Cluster-3923.0, Cluster-4296.0, Cluster-2665.0, and Cluster-3774.0 decreased expression) expressed differently (*p* < 0.05) between MeJA treatment MD33 and control MD33 (Figure 6 and Supplementary Table 1). The primary molecular functions were hydrolase and methyltransferase activities.

### Differentially Expressed Genes Related to Transcription Factors

Various Transcription Factors (TFs) were reported to participate in the biosynthesis of secondary metabolites in plants. In this study, 484 putative TF genes belonging to 31 major TF families were identified, and 17 genes belonging to 11 major TF families were analyzed (Figure 6). The Zn-clus (six genes) and the zinc finger protein contained the maximum members. Among these TF genes, three Zn-Clus genes, one SET gene, one C3H gene, and 1 GNAT gene were found to be up-regulated in the MeJA treatment MD33 group (Table 1).

### q-RT PCR Validation of Altered Genes

**FIGURE 7:**
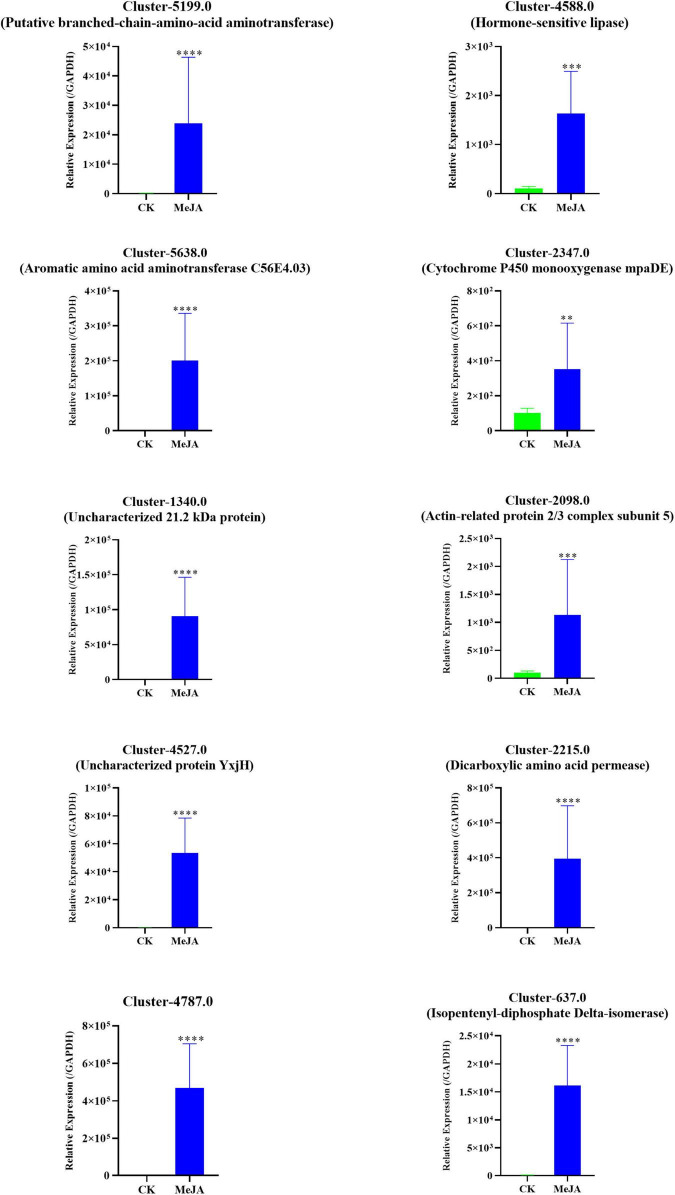
qRT-PCR verification of the RNA-seq data of 10 selected DEGs in the MeJA treatment group and Control. The related expression levels of the key genes involved in the MeJA-induced accumulation of alkaloids in MD33. The expression level of MeJA changed significantly between MeJA and control group. ^**^p< 0.01, ^***^p < 0.001, ^****^p < 0.0001.

A qRT-PCR assay with independent samples from the control and MeJA treatment groups was used to verify the expression changes of several key genes involved in the dendrobine biosynthesis pathway. In total 10 genes, including one MVA pathway genes, two P450 family genes, two methyltransferase genes, three aminotransferase genes, and two TF genes, were selected to confirm the RNA-seq data. The expression levels of these selected genes were consistent with the RNA-seq results (Figure 7 and Supplementary Table 5).

## Discussion

The primary active element in *Dendrobium nobile* is dendrobine, which is classified as a sesquiterpenoid alkaloid or terpenoid indole alkaloid (TIA) (Wang et al., 2020). Alkaloids were identified and reported for the first time in *Dendrobium* in 1932 (Suzuki et al., 1932; Chen and Chen, 1935). Recent studies have shown that the alkaloids found in *Dendrobium nobile* and *Dendrobium officinale* have significant antioxidant and pharmacological activities (Xu et al., 2017; Huang et al., 2019). As a common elicitor, MeJA was widely used in *D. nobile* and *D. officinal* to induce the accumulation of alkaloids (Chen et al., 2019; Zhang et al., 2022). Furthermore, MeJA can also promote the production of secondary metabolites in parasites (Chen et al., 2022) and fungi (Liang et al., 2015; Xu et al., 2021). However, the mechanism by which MeJA induces alkaloid accumulation in dendrobine-producing fungi has not been reported.

Given the same sesquiterpene backbone of these alkaloids, it was previously assumed that dendrobine-type alkaloids had comparable production routes. Dendrobine was produced using aminotransferases and methyltransferases after the sesquiterpene skeleton was generated (Yuan et al., 2018). Cytochromes P450s (CYP450s) play a critical role in the biosynthesis of sesquiterpene alkaloids (dendrobine) (Coon, 2005; Mou et al., 2021). Numerous CYP450s have been identified in *D. officinale* (Guo et al., 2013; Chen et al., 2019), *D. nobile* (Jin-Ling et al., 2017; Li et al., 2017), and *D. huoshanense* (Yuan et al., 2018). These were shown to be associated with many dendrobine synthesis pathways. For instance, CYP4 and CYP71 may catalyze monooxygenase and hydroxylation processes, respectively (Jin-Ling et al., 2017). Four putative genes (Cluster-4787.0, Cluster-514.0, Cluster-5538.0, and Cluster-5724.0) were identified in our RNA-seq data from three clans: CYP526, CYP65, and CYP531. Clan CYP65 was found to be the most abundant with 56 protein entries in *Trichoderma*, CYP526 was found to participate in secondary metabolic processes (Sonia et al., 2018) and CY (Moktali et al., 2012). This data suggests a novel function for the CYP526 and CYP65 clans in dendrobine accumulation. In this study, we found three MeJA-induced aminotransferase up-regulated genes and 15 MeJA-induced methyltransferase up-regulated genes (Chen et al., 2019). These genes have been annotated to play a role in the dendrobine biosynthesis pathway in *T. longibrachiatum* MD33.

There is increasing evidence that TFs are involved in the biosynthesis of various alkaloids, including bHLH, ERF, Orca, Zct, Gbf, and WRKY (Goklany et al., 2013). Some of these alkaloids exhibit Jasmonate reactivity, suggesting that the JA signaling cascade is involved in alkaloid production (Goklany et al., 2013; Yamada and Sato, 2013). The Fungal Transcription Factors Database FTFD^1^ contains information on 61 TF families, including bHLH, bZIP, C2H2 zinc finger, Zn2Cys6, and the zinc finger type GATA. These TFs have been identified in *T. atroviride*, *T. virens*, and *T. reesei* (Qian et al., 2021), and are involved in amino acid and vitamin synthesis, carbon and nitrogen metabolism, meiosis, and morphogenesis (Macpherson et al., 2006). Following MeJA treatment, many TFs were up-regulated, including zn-clus, SET, C3H, and GNAT. These differentially expressed TFs showed that they may play a role in dendrobine production.

## Conclusion

A total of 7,067 DEGs were identified in this study, od which some were linked to potential alkaloid biosynthesis pathway in *Trichoderma longibrachiatum* MD33. Unlike plants, the MVA pathway only regulates genes of the MD33 gene family. This indicates that MeJA treatment provides precursors for production of alkaloids. Additionally, multiple MeJA-induced P450 family genes, aminotransferase genes, and methyltransferase genes were identified, offering several prospective candidates for elucidating the probable alkaloid biosynthesis pathway of *T. longibrachiatum* MD33. These findings contribute to the understanding of the regulatory mechanism underlying MeJA-induced alkaloid accumulation in *T. longibrachiatum* MD33.

## Data Availability Statement

The RNA-seq used in this study have been deposited in the Sequence Read Achieve (SRA) of the NCBI database under the BioProject accession number: PRJNA809435.

## Author Contributions

QJ, LW, HJ, and SS performed the experiments. JC designed and supervised the project. XQ helped for the data curation. FS and LJ arranged the materials for experiments. QJ, LW, and SS wrote and edited the manuscript. All authors have read and agreed to the published version of the manuscript.

## Conflict of Interest

The authors declare that the research was conducted in the absence of any commercial or financial relationships that could be construed as a potential conflict of interest.

## Publisher’s Note

All claims expressed in this article are solely those of the authors and do not necessarily represent those of their affiliated organizations, or those of the publisher, the editors and the reviewers. Any product that may be evaluated in this article, or claim that may be made by its manufacturer, is not guaranteed or endorsed by the publisher.

## Footnotes

1
http://ftfd.snu.ac.kr/
